# MEDICAL VERSUS FINANCIAL RISKY DECISION MAKING IN OLDER AND YOUNGER ADULTS

**DOI:** 10.1093/geroni/igad104.0095

**Published:** 2023-12-21

**Authors:** Colleen Frank, Thorsten Pachur

**Affiliations:** Center for Vital Longevity, UT Dallas, Dallas, Texas, United States; Technical University of Munich, Munich, Bayern, Germany

## Abstract

While risky decision making is often tested using monetary outcomes in the laboratory, many decisions in the real-world elicit considerable levels of affect––for example, when deciding about a medical treatment that has bodily consequences, such as side effects to a medication. Previous research suggests that choices systematically diverge between these more “affect-rich” medical decision problems and economically equivalent “affect-poor” monetary problems, leading to what is called an ‘affect gap’ in risky choice. However, it is unknown to what extent this pattern holds in older adults, who make some of the most consequential medical and financial decisions among the population, and for whom it has been hypothesized that affect may be a more important guide. Thus, in the present, preregistered study, we examined age-related differences in the affect gap of risky decision making. We compared decision quality and risk attitude between affect-rich (medical side effects) and structurally identical affect-poor (monetary losses) decisions in 100 older (aged 65-80, M=69.7) and 100 younger (aged 18-29, M=23.5) adults. In line with previous findings, we found that individuals were more risk-averse (Odds Ratio (OR) = 1.52) and made worse quality decisions (OR = 2.13) for affect-rich, compared to affect-poor, problems and this did not differ between younger and older adults. Follow-up work using computational modeling will reveal if decision strategy differed between age groups. These findings indicate that the affect gap is also present in older adults, which has serious implications for risk communication in medical decision making

